# The diverse effects of pathogenic point mutations on ion channel activity of a gain-of-function polycystin-2

**DOI:** 10.1016/j.jbc.2023.104674

**Published:** 2023-04-05

**Authors:** Yan Wang, Zhifei Wang, Mahmud Arif Pavel, Courtney Ng, Parul Kashyap, Bin Li, Tiago D.C. Morais, Gabriella A. Ulloa, Yong Yu

**Affiliations:** Department of Biological Sciences, St. John’s University, Queens, New York, USA

**Keywords:** ion channel, transient receptor potential channels (TRP channels), kidney, mutant, protein structure, genetic disease, polycystin-2, gain-of-function

## Abstract

Autosomal dominant polycystic kidney disease is caused by mutations in *PKD1* or *PKD2* genes. The latter encodes polycystin-2 (PC2, also known as TRPP2), a member of the transient receptor potential ion channel family. Despite most pathogenic mutations in *PKD2* being truncation variants, there are also many point mutations, which cause small changes in protein sequences but dramatic changes in the *in vivo* function of PC2. How these mutations affect PC2 ion channel function is largely unknown. In this study, we systematically tested the effects of 31 point mutations on the ion channel activity of a gain-of-function PC2 mutant, PC2_F604P, expressed in *Xenopus* oocytes. The results show that all mutations in the transmembrane domains and channel pore region, and most mutations in the extracellular tetragonal opening for polycystins domain, are critical for PC2_F604P channel function. In contrast, the other mutations in the tetragonal opening for polycystins domain and most mutations in the C-terminal tail cause mild or no effects on channel function as assessed in *Xenopus* oocytes. To understand the mechanism of these effects, we have discussed possible conformational consequences of these mutations based on the cryo-EM structures of PC2. The results help gain insight into the structure and function of the PC2 ion channel and the molecular mechanism of pathogenesis caused by these mutations.

Autosomal dominant polycystic kidney disease (ADPKD), one of the most common genetic diseases in humans, is caused by mutations in either *PKD1* or *PKD2*, two genes encoding polycystin-1 (PC1) and polycystin-2 (PC2, or TRPP2) protein, respectively (1, 2, 3, 4). PC1 is a member of the polycystic kidney disease proteins family, which has 11 transmembrane domains, a large extracellular N terminus, and a relatively short intracellular C terminus (1, 5, 6). In contrast, PC2 protein belongs to the transient receptor potential (TRP) cation channel superfamily polycystin subfamily (TRPP) (7). Similar to the other TRP proteins, PC2 has six transmembrane domains and intracellular N and C termini (8, 9). The last six transmembrane domains of PC1 share sequence and structural similarity with the transmembrane domains of PC2.

Like all other TRP proteins, PC2 is involved in tetramer assembling. In the absence of PC1, PC2 forms a homotetramer and functions as a nonselective cation channel (10, 11, 12, 13). In the presence of PC1, three PC2 assemble with one PC1 into a heterotetramer through their C-terminal coiled-coil domain and a relatively large extracellular loop (14, 15, 16, 17, 18, 19). In this complex, both PC1 and PC2 function as ion channel pore-forming subunits (20). This extracellular loop, linking the first and second transmembrane domains in PC2 and the corresponding sixth and seventh transmembrane domains of PC1, is called tetragonal opening for polycystins (TOP) domain or polycystin-mucolipin domain (PMD) (21, 22, 23). The cryo-EM structures of the core fragments of the homomeric PC2 complex and the heteromeric PC1/PC2 complex, which includes the transmembrane domains and the TOP domains, have been resolved (14, 23, 24, 25). In both complexes, the TOP domains assemble into a donut-shaped tetramer, sitting on top of the transmembrane domains as a lid (14, 23, 24, 25). Due to its specific location in channel structures, TOP domains may also be involved in regulating channel gating and ion permeability as reported in TRPML1 (22).

The roles that the homomeric PC2 channel and the heteromeric PC1/PC2 channel play in ADPKD remain elusive, partially due to the lack of reliable activation mechanism of either homomeric PC2 or heteromeric PC1/PC2 channel, which has made their functional study very challenging. To help with this challenge, we developed gain-of-function (GOF) mutants of the PC2 and PC1/PC2 channels, which have provided capable platforms for channel function analysis of these channels (10, 20, 26, 27, 28). The GOF PC2 homomeric channel mutant, PC2_F604P, gave rise to a robust current when it was expressed in *Xenopus laevis* oocytes (10). In *in vivo* experiments, PC2_F604P, compared to wildtype PC2, more efficiently rescued the PC2 downregulation-induced morphological abnormalities in zebrafish embryos (10, 27). The cryo-EM structure of PC2_F604P shows that the F604P mutation leads to twisting and rotation of the bottom half of the S6 helix and opens the lower gate (27). The F604P mutation–induced conformational changes in the pore domain of PC2 are similar to what happens in the natural gating process of some other TRP channels such as TRPV, TRPML, and the homologous polycystin-L channels (27, 29, 30). Thus, we believe the structure of P2C_F604P should mimic the naturally gated wildtype PC2. This assumption set the foundation for using PC2_F604P in testing the effects of ADPKD pathogenic mutations.

Sequencing the PKD2 gene in ADPKD patients has identified more than 200 mutations that are likely pathogenic (Mayo Clinic ADPKD Database, http://pkdb.mayo.edu). Most of these mutations cause truncations, insertions, deletions, or frameshifts that lead to large changes in the protein sequence of PC2. Besides these mutations, 23 substitutions and three single-amino acid deletion mutations were found to be pathogenic (ADPKD Data Base, pkdb.mayo.edu, data by December 2021). Even though such point mutations cause small changes in protein sequences, they should have a large effect on the *in vivo* function of PC2 as they lead to ADPKD. Further investigation of their pathogenic mechanisms will enhance our understanding of the structure and function of PC2 and may also provide guides on early-onset ADPKD diagnostics and mutation-specific treatments. In this work, we studied how these 26 point pathogenic mutations, as well as five other point mutations, which have uncertain clinical significance, affect PC2 channel function by testing the effect of these mutations on the activity of the GOF PC2_F604P channel. Taking advantage of the reported cryo-EM, crystal, and NMR structures, we have also tried to link the functional results to potential conformational outcomes caused by these mutations.

## Results

### ADPKD pathogenic point mutations included in this study

**Figure 1:**
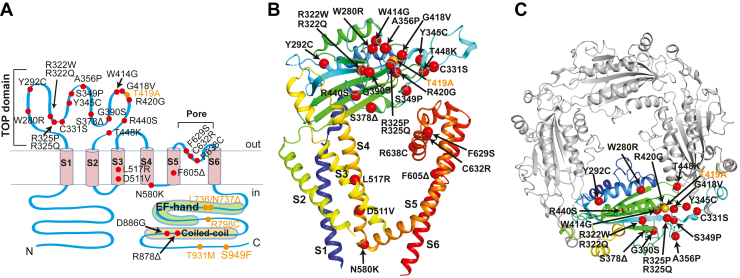
**Pathogenic point mutations in PC2**. *A*, membrane topology of PC2 channels, showing the location of the point pathogenic mutations tested in this study. The *red dots* indicated the 26 pathogenic mutations tested in this study, and the *orange dots* indicated another five point mutations included in this study that have uncertain clinical significance. *B*, 25 mutations were mapped on the side view of the cryo-EM structure of one subunit of PC2 (PDB: 5T4D) (25) The others are not visible on the current reported structures. Structure is colored in a *rainbow spectrum* with the N terminus in *blue* and the C terminus in *red*. The positions of the mutations are indicated with the residues’ alpha carbons shown in *spheres*. *C*, same as (*B*) with four subunits shown from a top view. Unless otherwise indicated, structural analysis in this study used the cryo-EM structure of the WT PC2 channel published by Shen *et al*. (25), instead of the cryo-EM structure of PC2_F604P (27), since the overall resolution of the former (3 Å) is much higher than that of the latter (3.5 Å). PC2, polycystin-2.

Among the 26 pathogenic point mutations, 17 are located on the extracellular TOP domain, which appears as a hotspot for ADPKD pathogenic mutations (Fig. 1, *A*–*C*); seven are mapped on transmembrane domains 3 to 6, a critical area associated with channel gating and ion permeability (Fig. 1, *A* and *B*); and two were found on the intracellular C-terminal tail of PC2 (Fig. 1*A*). In our tests, we have also included another five single or double amino acid substitution/deletion mutations that were reported in the ADPKD Database but were found to have uncertain clinical significance (Fig. 1*A*, labeled in *orange*). Four of them are in the C terminus of the channel, and one (T419A) is in the TOP domain. Among the 31 mutations, nine have been tested in our previous study (10). To give a comprehensive view of this topic, we tested these mutants again with the others. The new results of these mutations are consistent with those in the previous study.

### The majority of the TOP domain pathogenic mutations reduce the current of the GOF PC2_F604P channel

**Figure 2:**
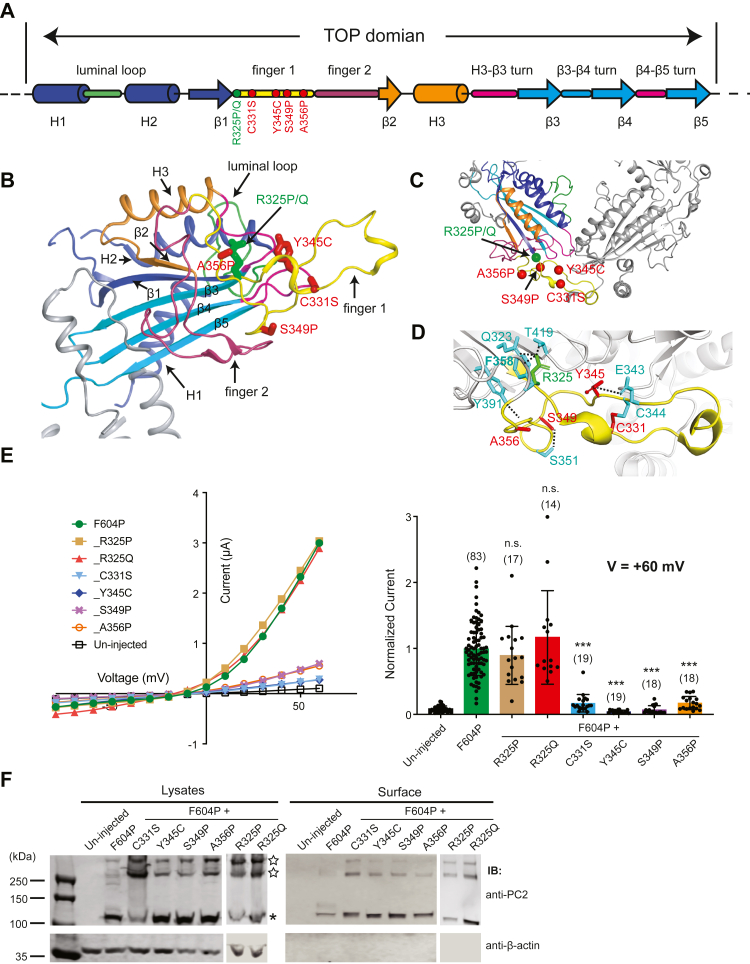
**Most mutations in finger 1 of the TOP domain significantly reducethechannel activity of PC2_F604P**. *A*, the diagram shows the structural features of the TOP domain of PC2. *Red dots* indicate the positions of the four finger 1 pathogenic mutations that significantly reduce the channel activity of PC2_F604P. *Green dot* indicates the position of the mutants R325P and R325Q, which did not significantly affect the channel activity. The same color codes are used in (*B* and *C*) and other figures. *B*, the tested mutations in finger 1 are shown with *sticks* on the side view of the cryo-EM structure of the TOP domain (25). *C*, *top* view of the TOP domains from two PC2 subunits, showing the positions of the tested mutations and that finger 1 is directly involved in TOP domain interaction. The positions of the mutations are indicated with the residues’ alpha carbons shown in *spheres*. *D*, structural details show the local interactions that mutated residues are involved. *Black dashed lines* indicate hydrogen bonds. C331 and C344 form a disulfide bond. *E*, representative I–V curves (*left*) and a scatter plot and bar graph (*right*) showing the currents of the indicated PC2 channels. The scatter plot and bar graph show the current sizes at +60 mV in a solution containing 100 mM NaCl, 2 mM CaCl_2_, and 2 mM Hepes, pH 7.5. Oocyte numbers for the bar graph are indicated in parentheses. The inward currents are inhibited by extracellular Ca^2+^ as previously reported (10). Data are presented as mean ± SD in bar graph (n.s.: not significant, ∗∗∗*p* < 0.001, Student’s *t* test). *F*, surface biotinylation followed by Western blot showing the expression levels of the indicated proteins in lysate and plasma membrane of oocytes. *Asterisk* indicates the monomer band of PC2 on the SDS-PAGE gel, while the *hollow stars* indicate the oligomers. Although at most of the time, the density of the oligomer bands can be random (may be due to variance in sample preparation), they are always heavier in the lysate sample of the mutant C331S (*left panel*). However, the oligomer bands in the surface sample are similar to that of the other mutants (*right panel*). PC2, polycystin-2; TOP, tetragonal opening for polycystins.

The lid-like extracellular TOP domain is composed of three α-helices (H1-3), five anti-parallel β sheets (β1-5), and two extended intersubunit interaction motifs called finger 1 and finger 2 (Fig. 2, *A* and *B*) (25) [named three-leaf clover extension 1 and 3 in (23)]. Finger 1 is a protruding hairpin-like structure that is essential for efficient association with another β-turn structure from the TOP domain of the neighboring subunit (23, 25). Mutations on finger 1 may disrupt the homotetrameric channel assembly. Six substitution mutations, R325P, R325Q, C331S, Y345C, S349P, and A356P are localized on finger 1 and thus at the interface between TOP domains from two neighboring subunits (Fig. 2*C*). To determine their effects on channel activity, we introduced these mutations into the GOF PC2_F604P channel, expressed them in *X. laevis* oocytes, and tested their channel function with the two-electrode voltage clamp (TEVC) method. The results show that the two R325 mutations, R325P and R325Q, did not change channel current, while the other four mutants, C331S, Y345C, S349P, and A356P, lead to largely reduced, even abolished current of PC2_F604P channel (Fig. 2*E*). Western blot shows that PC2_F604P and the additional mutants were all expressed well in oocytes (Fig. 2*F*, left panel). By purifying the channel proteins on the plasma membrane with a surface biotinylation method, we found that PC2_F604P and all the new mutant channels, including the four that have significantly low channel activity, have good expression on the plasma membrane (Fig. 2*F*, right panel). Thus, the reduced current of the four mutants mentioned above most likely is not caused by poor expression or trafficking of the channel proteins in oocytes.

Analyzing the cryo-EM structure of finger 1 gives us hints on the possible molecular mechanism of the effects of these mutations. Among the five affected residues, Y345 is directly involved in the assembly of the TOP domain tetramer by forming a hydrogen bond with N432 in the TOP domain from the neighboring subunit (25). All the other four residues are not directly involved in the interaction between TOP domains. However, most of them play key roles in maintaining the structure of the finger 1 (Fig. 2*D*). C331 forms a disulfide bond with C344, which fixes the overall shape of finger 1 (Fig. 2*D*) (23, 25). A previous study has shown that this disulfide bond is essential for PC2 channel function (31). Thus, the C331S mutation will break this interaction and lead to the deformation of finger 1. Since Y345 is close to C331 and C344, Y345C mutation may potentially disturb the correct disulfide bond formation between C331 and C344 by interacting with one of them. C349P and A356P will lead to conformational change at the ending part of finger 1 (Fig. 2*D*) and abolish the interaction between the backbone of A356 with Y391 in the second β sheet (β2). R325 is located at the starting point of finger 1 and is involved in interaction with Q323 in β1, T419 in β3, and P358 at the end of finger 1 (Fig. 2*D*). Since adding either R325P or R325Q mutations did not change the activity of the PC2_F604P channel, R325 and its interactions are not essential for the channel function of PC2_F604P.

**Figure 3:**
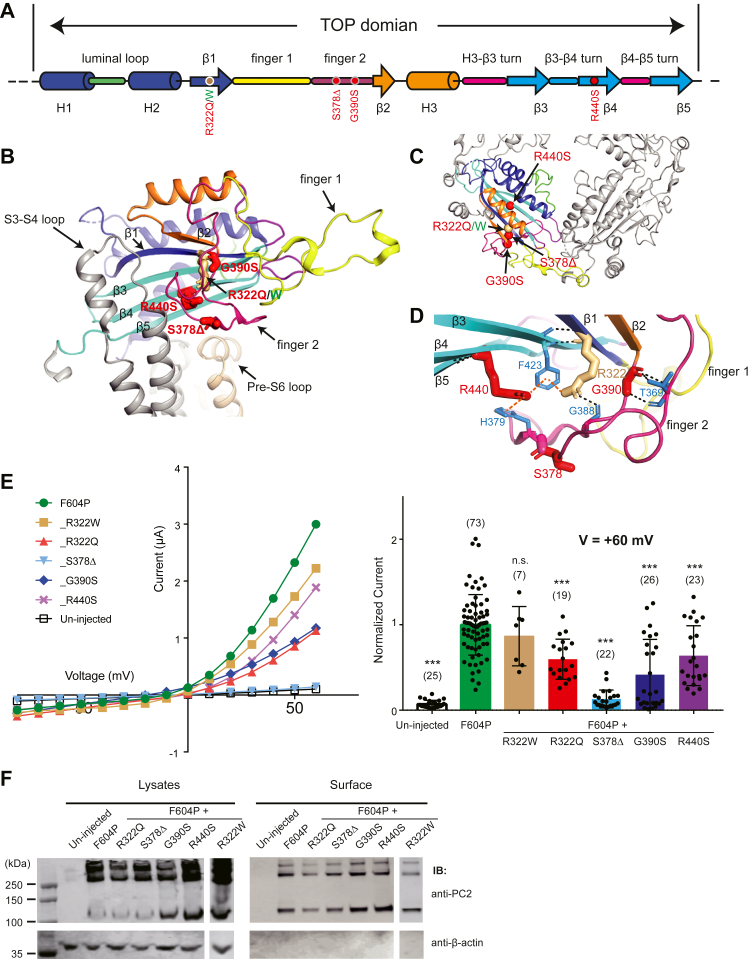
**Most mutations in finger 2 and near regions of the TOP domain significantly reduce channel activity of PC2_F604P**. *A*, the diagram shows the structural features of the TOP domain of PC2. *Red dots* indicate the positions of the three pathogenic mutations that significantly reduce the channel activity of PC2_F604P. The *brown dot* indicates the positions of mutations R322Q and R322W. *B*, the tested mutations are shown on the side view of the cryo-EM structure of the TOP domain (25). *C*, top view of the TOP domains from two PC2 subunits, showing the positions of the tested mutations. *D*, structural details show the local interactions that R322, S378, G390, and R440 are involved. *Black and orange dashed lines* indicate hydrogen bonds and cation-π interaction, respectively. *E*, representative I–V curves (*left*) and a scatter plot and bar graph (*right*) showing the currents of the indicated PC2 channels. The scatter plot and bar graph show the current sizes at +60 mV in a solution containing 100 mM NaCl, 2 mM CaCl_2_, and 2 mM Hepes, pH 7.5. Oocyte numbers for the bar graph are indicated in parentheses. Data are presented as mean ± SD in bar graph (n.s.: not significant, ∗∗∗*p* < 0.001, Student’s *t* test). *F*, surface biotinylation followed by Western blot showing the expression levels of the indicated proteins in lysate and plasma membrane of oocytes. PC2, polycystin-2; TOP, tetragonal opening for polycystins.

The mutation sites R322 (with mutations R322Q and R322W), S378 (S378Δ), G390 (G390S), and R440 (R440S) are in β1, finger 2, and β4, respectively. However, they stay in close proximity in the 3D structure, and all face toward the bottom of the TOP domain (Fig. 3, *A* and *B*). Therefore, we tested them as a group. In the cryo-EM structure of PC2, finger 2 interacts with the outer pore pre-S6 loop of the neighboring subunit and may be directly involved in regulating pore structure and ion permeation (Fig. 3, *B* and *C*) (25). Beta sheets 1 and 4 (β1 and β4) are essential for the overall structure of the TOP domain and interact with the loop between S3 and S4 (Fig. 3, *B* and *C*) (25). The turn between β3 and β4 (β3- β4 turn) interacts with finger 1 from the neighboring TOP domain and is essential for TOP tetramer assembly (25). Our recording results show that all mutations, except for R322W, significantly reduce the PC2_F604P current (Fig. 3*E*). Among them, S378Δ only gave rise to very small currents (Fig. 3*E*). Surface biotinylation results show that all mutants, including the S378Δ, have relatively good overall and surface expression (Fig. 3*F*), suggesting that the reduction of channel currents may not simply be caused by reduced protein expression.

Analysis of the structural details demonstrates that R322 is involved in stabilizing the β-sheets structure *via* backbone hydrogen bonds (Fig. 3*D*). It also bridges the interaction between the β-sheets and finger 2 by forming cation-π interaction with F423 on β3 and a hydrogen bond with G388 in finger 2 (Fig. 3*D*). R322W mutation probably can preserve this bridging by forming π-π stacking interaction with F423, thus keeping the channel function. On the other hand, R322Q mutation will lead to the loss of these interactions. S378 seems to not be involved in any direct interaction with the surrounding residues (Fig. 3*D*). However, the lack of this residue in the S378Δ mutant will lead to a dramatic change in the local conformation. G390 has intensive hydrogen bond interaction with T369 in finger 2 (Fig. 3*D*). G390S will interfere with these interactions and lead to distortion of the structure of finger 2. R440 plays a similar role as R322 in bridging the interaction between finger 2 and the β sheets by forming cation-π interactions with both F423 on β3 and H379 in finger 2 (Fig. 3*D*), and R440S mutation will abolish these interactions.

**Figure 4:**
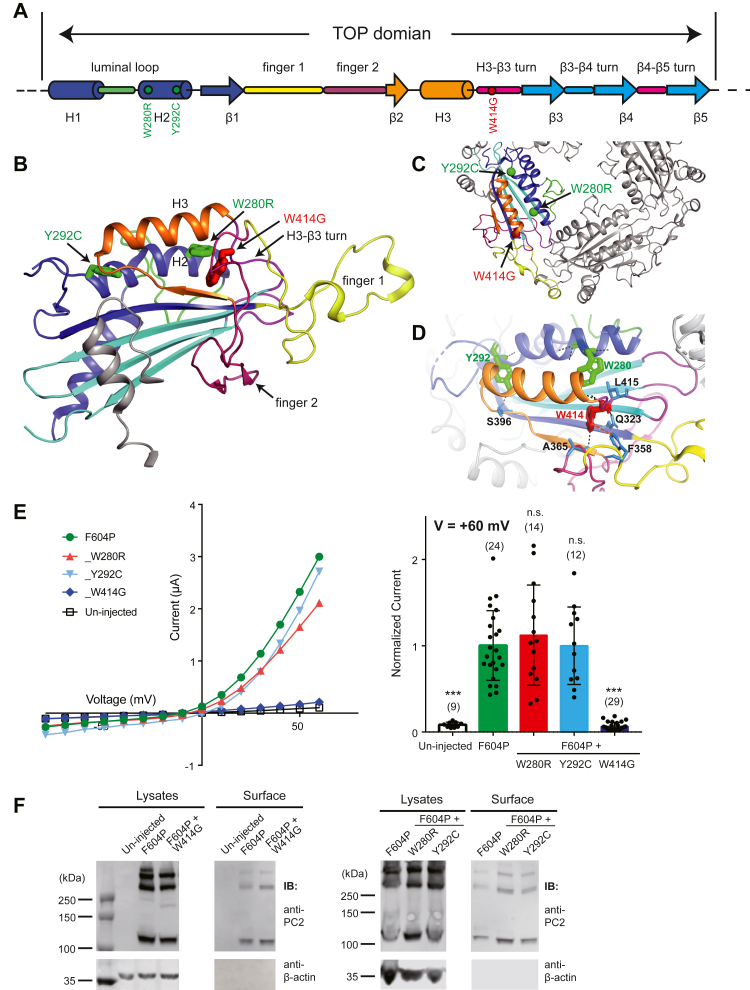
**Effects of three pathogenic mutations in the hydrophobic core of the TOP domain.***A*, the diagram shows the structural features of the TOP domain of PC2. The *red dot* indicates the position of mutation W414G. *Green dots* indicate the positions of W280R and Y292C. *B*, the tested mutations are shown on the side view of the cryo-EM structure of the TOP domain (25). *C*, top view of the TOP domains from two PC2 subunits, showing the positions of the tested mutations. *D*, structural details show the local interactions that W280, Y292, and W414 are involved. *Black* and *orange dashed lines* indicate hydrogen bonds and π-stacking interaction, respectively. *E*, representative I–V curves (*left*) and a scatter plot and bar graph (*right*) showing that the currents of the indicated PC2 channels. The scatter plot and bar graph show the current sizes at +60 mV in a solution containing 100 mM NaCl, 2 mM CaCl_2_, and 2 mM Hepes, pH 7.5. Oocyte numbers for the bar graph are indicated in parentheses. Data are presented as mean ± SD in bar graph (n.s.: not significant, ∗∗∗*p* < 0.001, Student’s *t* test). *F*, surface biotinylation followed by Western blot showing the expression levels of the indicated proteins in lysate and plasma membrane of oocytes. The samples in the *left panel* were run together with the samples in the *left panel* of Fig. 5*F* in our experiment and are presented and discussed separated in two figures based on the mutations’ locations in the cryo-EM structure. Thus, the first two lanes (“uninjected” and “F604P”) in both lysates and surface samples, as the negative and positive controls, were used in both Figs. 4*F* and 5*F*. PC2, polycystin-2; TOP, tetragonal opening for polycystins.

We next checked three mutations, W280R, Y292C, and W414G, that are located between the two extracellular-facing helices (helix 2 and 3) and the β-sheets in the TOP domain (Fig. 4, *A*–*C*). W280 and Y292 are in helix 2, while W414 is in the loop turn between helix 3 and β3. They are hydrophobic residues and play critical roles in stabilizing the hydrophobic core of the TOP domain structure. Our recording data show that while the W414G mutation completely abolishes the current of PC2_F604P, W280R, or Y292C mutations led to no change in the PC2_F604P channel current (Fig. 4*E*). All mutants, including W414G, have good overall and plasma membrane expression as detected by surface biotinylation (Fig. 4*F*). When checking the local interactions where the three residues are involved in, we found that the side chain of W280 has loose hydrophobic interaction with L415, while that of Y292 is only involved in forming one hydrogen bond with S396 (Fig. 4*D*). The lack of intensive interaction of these two residues may explain why PC2_F604P-W280R and PC2_F604P-Y292C still have relatively normal channel function (Fig. 4*E*). In contrast, the side chain of W414 forms hydrogen bonds with A365 on finger 2 and π-stacking interaction with F358 on finger 1 (Fig. 4*D*). The W414G mutation will abolish these interactions and destabilize the confirmation of both fingers 1 and 2.

**Figure 5:**
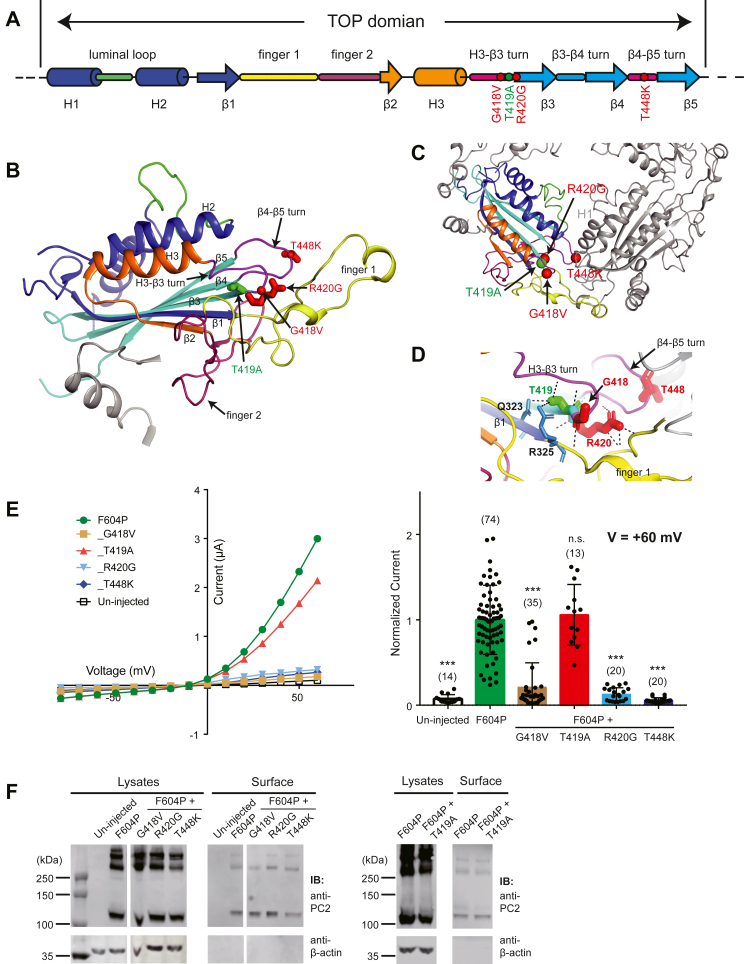
**Variant effects caused by four mutations on the side of the TOP domain**. *A*, the diagram shows the structure features of the TOP domain of PC2. *Red dots* indicate the positions of three mutations which significantly reduce the channel activity of PC2_F604P. *Green dots* indicate the position of the T419A which does not reduce channel activity. T419A is indicated as a mutation that causes uncertain clinical significance in ADPKD Database (pkdb.mayo.edu). *B*, the tested mutations are shown on the side view of the cryo-EM structure of the TOP domain (25). *C*, top view of the TOP domains from two PC2 subunits, showing the positions of the tested mutations. *D*, structural details show local interactions that G418, T419, R420, and T448 are involved. *Black dashed lines* indicate hydrogen bonds. *E*, representative I–V curves (*left*) and a scatter plot and bar graph (*right*) showing the currents of the indicated PC2 channels. The scatter plot and bar graph show the current sizes at +60 mV in a solution containing 100 mM NaCl, 2 mM CaCl_2_, and 2 mM Hepes, pH 7.5. Oocyte numbers for the bar graph are indicated in parentheses. Data are presented as mean ± SD in bar graph (n.s.: not significant, ∗∗∗*p* < 0.001, Student’s *t* test). *F*, surface biotinylation followed by Western blot showing the expression levels of the indicated proteins in lysate and plasma membrane of oocytes. The samples in the *left panel* were run together with the samples in the *left panel* of Fig. 4*F* in our experiment and are presented and discussed separated in two figures based on the mutations’ locations in the cryo-EM structure. Thus, the first two lanes (“un-injected” and “F604P”) in both lysates and surface samples, as the negative and positive controls, were used in both Figs. 4*F* and 5*F*. ADPKD, autosomal dominant polycystic kidney disease; PC2, polycystin-2; TOP, tetragonal opening for polycystins.

The H3-β3 turn and β4-β5 turn are close to each other in the cryo-EM structure of PC2, and they interact with the H1 helix, H1-β3 turn, and β3-β4 turn from the adjacent subunit, forming a significant portion of the binding interface between two TOP domains (Fig. 5, *A*–*C*) (25). Thus, the structure of these two turns is critical for TOP domain tetramer assembly. In this area, three pathogenic mutations (G418V, R420G, and T448K) and one mutation with uncertain significance (T419A) are included in the PKD database. In our recordings, we found that all three pathogenic mutations abolished the current of PC2_F604P, while T419A led to no effect to channel current (Fig. 5*E*). All mutations, including the three pathogenic mutations that have significantly small channel currents, have good overall and plasma membrane expression as detected by surface biotinylation (Fig. 5*F*). We then checked the interactions that these residues are involved in the cryo-EM structure (Fig. 5*D*) (25). We found that the side chain of R420 is intensively involved in forming hydrogen bonds with backbone and residues in finger 1 and the β4-β5 turn and plays key roles in maintaining the local confirmation (Fig. 5*D*). Although not many direct interactions were found formed by the side chains of G418 and T448, G418V will most likely reduce the flexibility of the H3-β3 turn, and T448K will dramatically change the charge of the local environment and affect the interaction with the neighboring TOP domain. Consistently, we previously found that the T448K mutation reduced the interaction between PC2 TOP domains (19). In the cryo-EM structure, the side chain of T419 is involved in forming hydrogen bonds with Q323 and R325 at β1-finger 1 junction and with the backbone of the H3-β3 turn (Fig. 5*D*). Thus, it plays a role in maintaining the local confirmation. Since T419A does not change PC2_F604P current, we assume that either these interactions are not critical, or the alanine mutation preserves most of these interactions.

Together, by performing TEVC recording, we show that most of the TOP domain pathogenic mutations significantly decrease or completely abolishe the channel activity, although some others do not have an obvious effect on the current of the GOF PC2_F604P channel. Since these mutations have a relatively good expression on the plasma membrane, the overall structure of PC2 must have been preserved in these mutant channels. Thus, we hypothesize that the function reduction is due to the local conformational changes or defects in TOP domain assembly caused by these mutations.

### Pathogenic mutations in the transmembrane domains abolish the PC2-F604P channel activity

**Figure 6:**
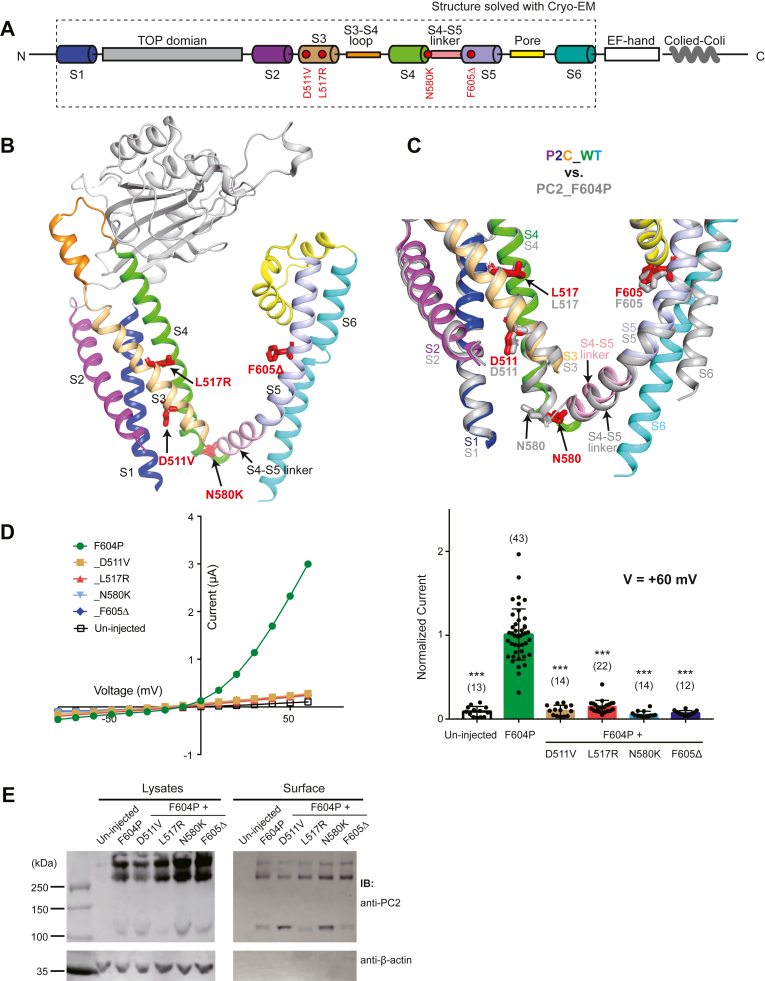
**Pathogenic mutations in transmembrane domains abolish channel activity of PC2_F604P**. *A*, the diagram shows the structural features of PC2. The structure of the fragment included in the *dotted box* has been resolved with cryo-EM. *Red dots* indicate the positions of four mutations tested in this figure. *B*, the tested mutations are shown on the side view of the cryo-EM structure of PC2 (25). *C*, the structures of WT PC2 (25) and PC2_F604P (27) are aligned to show the conformational changes of the S4-S5 linker, S5, S6, as well as the four mutated residues, in PC2_F604P. *D*, representative I–V curves *(left*) and a scatter plot and bar graph (*right*) showing the currents of the indicated PC2 channels. The scatter plot and bar graph show the current sizes at +60 mV in a solution containing 100 mM NaCl, 2 mM CaCl_2_, and 2 mM Hepes, pH 7.5. Oocyte numbers for the bar graph are indicated in parentheses. Data are presented as mean ± SD in bar graph (∗∗∗*p* < 0.001, Student’s *t* test). *E*, surface biotinylation followed by Western blot showing the expression levels of the indicated proteins in lysate and plasma membrane of oocytes. PC2, polycystin-2.

Four pathogenic single amino acid mutations, D511V, L517R, N580K, and F605Δ, were found in the transmembrane helices of PC2 (Fig. 6, *A* and *B*). Similar to other TRP channels, each PC2 subunit has six transmembrane helices (S1-S6). The first four (S1-S4) forms a domain that is structurally like the voltage sensor domain (VSD) of canonical voltage-gated cation channels, although only two (K572 and K575) of four gating charges are preserved in S4 of PC2 (25). The last two transmembrane helices (S5 and S6) form the pore domain, which is directly involved in forming the ion-conducting pore of PC2 and all other TRP channels. In voltage-gated channels, the link between S4 and S5 (S4-S5 linker) is essential for conducting the voltage sensing by VSD to channel gating. The S4-S5 linker and first half of S5 also play key roles in the gating of TRP channels since multiple mutations in this region have been found to lead to the GOF effect of many TRP channels (32). Previously, we generated a GOF PC2 channel by introducing the F604P mutation (10). F604P is in the first half of S5 and is in the core of a cluster of hydrophobic interactions between S5 and S6 (10, 23). Although the overall cryo-EM structure of PC2_F604P is similar to that of the WT PC2, significant structural shifts happen at the S4-S5 linker and S5, most likely due to the bend of S5 and the weakened interaction between S5 and S6 caused by F604P mutation (Fig. 6*C*) (27). At the same time, twisting and bending movements of distal S6, induced by the transition of a π-helix structure in the middle of S6 to α-helix, lead to the opening of the lower gate of the channel (Fig. 6*C*) (27).

Our results show that all four pathogenic mutations found in the transmembrane domains abolished the current of PC2_F604P (Fig. 6*D*). In our surface biotinylation experiments, all four pathogenic mutations showed significant expression on plasma membrane (Fig. 6*E*). Although we noticed some variation on the overall and surface expression between that of PC2_F604P and some of the new mutants (Fig. 6*E*), the almost complete absence of channel activity in these four mutations are clearly not caused by loss of surface expression.

The first two mutations D511V and L517R are in S3. Similar to our results, D511V mutation has been previously shown in several studies to not affect the expression and trafficking of PC2 expressed in cultured cells but abolished PC2 function, including its channel activity (10, 11, 33, 34, 35, 36), although another one reported that D511V reduces the stability of exogenously expressed PC2, which leads to lower expression of PC2 (37). In the cryo-EM structure, D511 was found to directly interact with both K572 and K575 to stabilize both S3 and S4 (25). Thus, mutation D511V will most likely disturb the VSD structure. Similarly, switching from a hydrophobic residue to a positively charged residue on S3, in the case of the L517R mutation, may abolish the channel activity due to a similar reason. N580 is located at the junction of S4 and the S4-S5 linker (Fig. 6*B*). The latter has a significant structural shift in PC2_F604P structure compared to that of WT, which leads to a roughly 180° turn of the N580 side chain (Fig. 6*C*). The N580K mutation will restrict the proper structure shift in this region. As for F605Δ, a significant conformational change around F604P is expected due to this mutation. Thus, both N580K and F605Δ mutations seem to abolish channel activity by directly affecting the GOF effect of the F604P mutation. However, due to the essential role of the S4-S5 linker and the first half of S5 in TRP channel gating, these two mutations most likely will also affect the gating of WT PC2. The D511V, L517R, and N580K may also interrupt a potential PIP2 binding in this region, which was proposed in a previous study (38).

### Pathogenic mutations in the outer pore region abolish the PC2_F604P channel function

**Figure 7:**
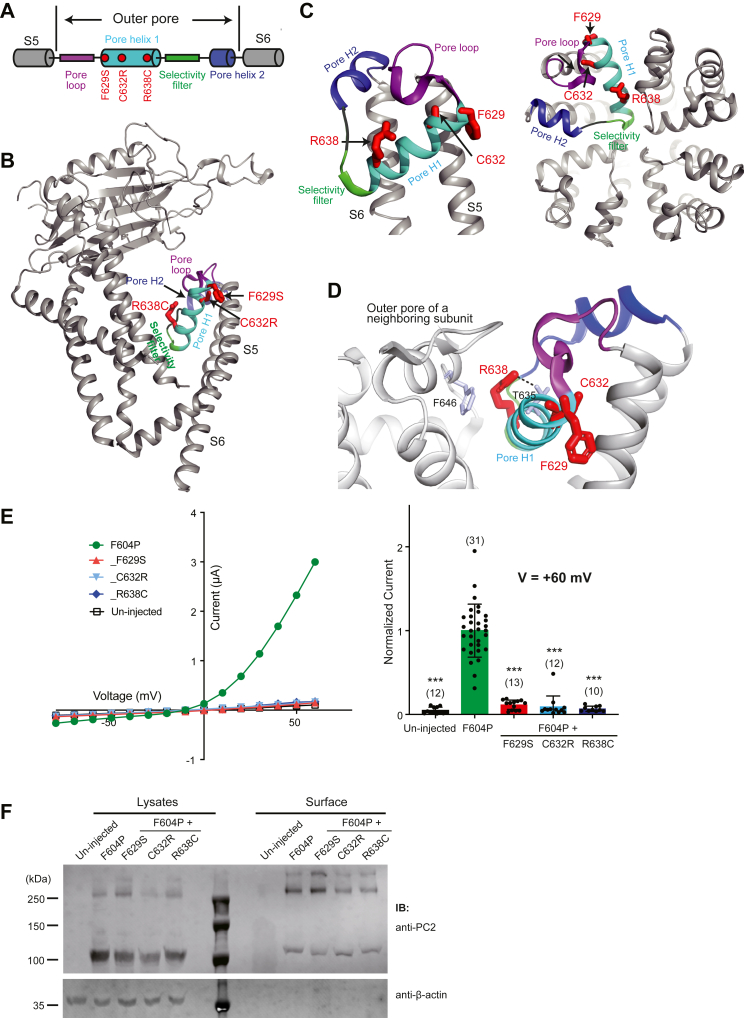
**Pathogenic mutations in the outer pore region abolishthechannel activity of PC2_F604P.***A*, the diagram shows the structural features of the outer pore region of PC2. *Red dots* indicate the positions of three pore helix 1 mutations tested in this figure. *B*, the tested mutations are shown on the side view of the cryo-EM structure of PC2 (25). *C*, detailed views of the outer pore region from the side (*left*) and the *top* (*right*), showing the positions of the tested mutations. *D*, structural details to show that R638 is essential for stabilizing the structure of pore helix 1 by forming a cation-π interaction with F646 on a neighboring subunit and a hydrogen bond with T635 in pore helix 1. *E*, representative I–V curves (*left*) and a scatter plot and bar graph (*right*) showing that the currents of the indicated PC2 channels. The scatter plot and bar graph show the current sizes at +60 mV in a solution containing 100 mM NaCl, 2 mM CaCl_2_, and 2 mM Hepes, pH 7.5. Oocyte numbers for the bar graph are indicated in parentheses. Data are presented as mean ± SD in bar graph (∗∗∗*p* < 0.001, Student’s *t* test). *F*, surface biotinylation followed by Western blot showing the expression levels of the indicated proteins in lysate and plasma membrane of oocytes. PC2, polycystin-2.

The last two transmembrane helices of PC2, S5 and S6, form the ion-conducting pore of the channel. The outer pore region of PC2, formed by the linker between S5 and S6, has a classic structure that contains the outer pore loop, the first pore helix (pore H1), a selectivity filter loop, and then the shorter second pore helix (pore H2) (Fig. 7, *A* and *B*) (23, 24, 25). There are three ADPKD pathogenic single amino acid mutations, F629S, C632R, and R638C, which lie in the pore H1 of the outer pore region (Fig. 7, *B* and *C*). In our functional test, all three mutations abolished the activity of PC2_F604P, even though they expressed well on plasma membrane (Fig. 7, *E* and *F*). We assume that all three mutations likely preclude ion permeability of the channel by disturbing the structure of the outer pore region. In the cryo-EM structure, R638 is essential for stabilizing the structure of pore helix 1 by forming a hydrogen bond with T635 in pore helix 1 and a cation-π interaction with F646 on a neighboring subunit (Fig. 7*D*) (25). C632 has also been previously shown to play a role in the assembly of PC2 (39).

### Pathogenic mutations in the C-terminal tail do not have large effect on the PC2_F604P channel function

**Figure 8:**
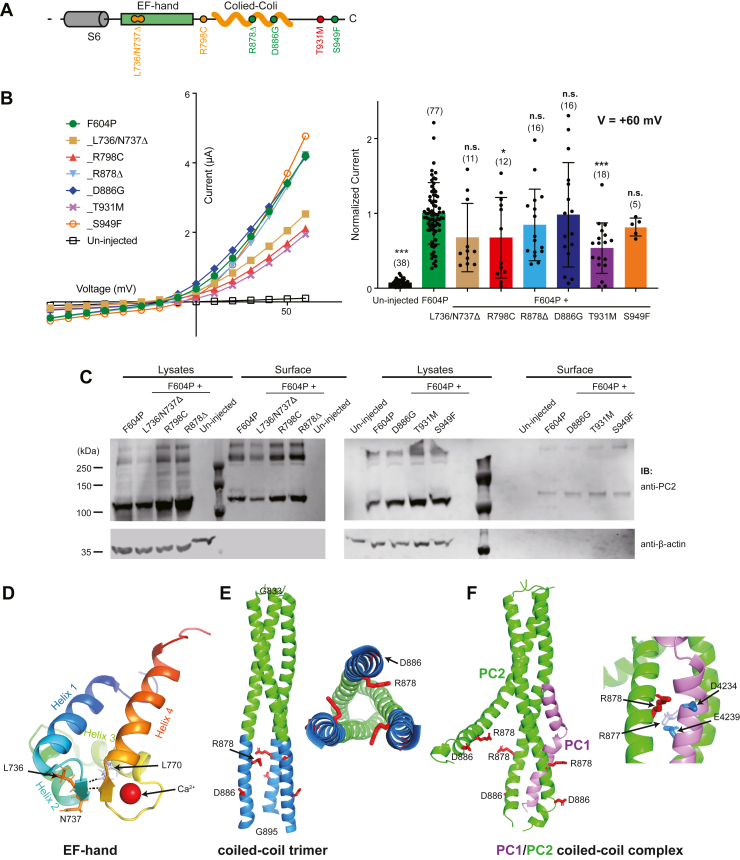
**Effects of the pathogenic mutations in C-terminal tail of PC2_F604P**. *A*, the diagram shows the structural features of the C-terminal tail of PC2. *Red and orange dots* indicate the positions of the mutations that significantly reduce the channel activity of PC2_F604P, while *green dots* indicate the positions of the mutations that do not significantly reduce channel activity. *B*, representative I–V curves (*left*) and a scatter plot and bar graph (*right*) showing the currents of the indicated PC2 channels. The scatter plot and bar graph show the current sizes at +60 mV in a solution containing 100 mM NaCl, 2 mM CaCl_2_, and 2 mM Hepes, pH 7.5. Oocyte numbers for the bar graph are indicated in parentheses. Data are presented as mean ± SD in bar graph (∗*p* < 0.05, ∗∗∗*p* < 0.001, Student’s *t* test). *C*, surface biotinylation followed by Western blot shows the expression levels of the indicated proteins in lysate and plasma membrane of oocytes. Results shown in two groups from two separate experiments. Although samples in the second group gave significant lower surface signal due to the usage of different batch of the surface biotinylation kit, it is clear that the new mutants had comparable surface expression as F604P in the same group. *D*, NMR structure of the EF-hand domain of PC2 showing the location of L736 and N737, as well as the hydrogen bonds (*dotted lines*) between L736 and L770 (PDB ID: 2Y4Q) (40). The single bound Ca^2+^ is shown as a *sphere* in *red*. *E*, side (*left*) and bottom (*right*) views of the crystal structure of the trimer formed by coiled-coil domains from three PC2 subunits (PDB ID: 3HRN) (16), showing the positions of the two tested mutations in this domain. *F*, *left*: structural model of the complex formed by three PC2 coiled-coil domains and one PC1 coiled-coil domain (15), showing the positions of the tested two mutations. *Right*: Structural details of the model to show the interaction between R877 and R878 on PC2 and D4234 and E4239 on PC1. PC2, polycystin-2; PC1, polycystin-1.

The intracellular C-terminal tail of PC2 has functional significance, as many ADPKD pathogenic mutations in PC2, such as N720X, R742X, and Y762X, result in truncation of this tail (ADPKD Database, pkdb.mayo.edu). The C-terminal tail of PC2 contains two significant structural elements: a Ca^2+^-binding EF-hand domain (N720-S795) and a coiled-coil domain (Y836-L894) (Fig. 8*A*). The structure of the EF-hand domain has been published (40, 41). However, the role that Ca^2+^ binding in this domain plays in regulating channel activity has been controversial (42, 43). The coiled-coil domain is involved in protein assembly. Two early studies demonstrated that coiled-coil domains in the C-terminal tail of PC1 and PC2 are involved in their association (17, 18). Later studies from our and the other labs refined the location of this coiled-coil domain to a Y836-L894 region and found that this domain forms a trimer in solution and plays a role in both homomeric assembly of PC2 and heteromeric assembly between PC1 and PC2 (15, 16, 44, 45, 46). Abolishing the coiled-coil trimer formation greatly weakens the homomeric assembly of PC2 and the heteromeric assembly between PC1 and PC2 (16, 23). Later evidence shows that the coiled-coil domains are not necessary for PC1 and PC2 assembly when proteins are overexpressed, possibly due to the interactions at the TOP and the transmembrane domains (14, 24, 25, 39, 45). However, these results do not rule out the possible critical role of the coiled-coil interaction in complex assembly when protein expression level is low *in vivo*.

In our experiments, we tested six mutations located in the C-terminal tail. Two of them, D866G and R878Δ, are likely pathogenic, while the other four, L736/N737Δ, R798C, T931M, and S949F, have uncertain significance according to the ADPKD Database (pkdb.mayo.edu) (Fig. 8*A*). Our results show that these mutations have a relatively moderate effect on PC2_F604P channel activity, except for T931M, which has about half the activity compared to PC2_F604P (Fig. 8*B*). When being checked for surface expression, all mutations showed a comparable amount of surface expression as PC2_F604P (Fig. 8*C*). Due to the limits of the experiential methods, we could not completely rule out the possibility that the subtle change in current sizes of R798C mutant is the result of small changes in surface expression. However, it is clear that the significant smaller current of the T931M mutant was not simply caused by defect in its surface expression.

Three cryo-EM structures of PC2 homotetramer and one of PC1/PC2 heterotetramer have been previously reported (14, 23, 24, 25). However, the intracellular N and C termini of PC2 are missing in all structures, due to either the truncated constructs used or the flexibility of the termini. Although structural information of R798, T931, and S949 are not available, L736 and N737 are seen in the NMR structures of the EF-hand domain (Fig. 8*D*) (40, 41), and D866 and R878 are in the crystal structure of the coiled-coil domain (Fig. 8*E*) (16). Unlike the canonical EF-hand domains, the one in PC2 contains only one Ca^2+^ binding site formed in the second helix-loop-helix motif, but not the first (Fig. 8*D*) (40, 41). Hydrogen bonds formed between L736 and L770 (Fig. 8*D*) connect the two antiparallel loops and stabilize the structure Ca^2+^ binding site. Thus, L736/N737Δ mutation will likely destabilize the Ca^2+^ binding. Here, we found that this mutation did not significantly reduce channel activity of PC2_F604P (Fig. 8*B*). This result is consistent with what was previously reported that abolishing the Ca^2+^-binding in the EF-hand domain does not change PC2 channel function in primary cilia and ER membrane (42).

We have previously solved a crystal structure of the PC2 coiled-coil domain trimer (16). In this structure, the N-terminal two thirds of the coiled-coil domain is tightly bundled together by hydrophobic interactions among the three helices, while the C-terminal one thirds of domain, from A873 to G895, splays open (Fig. 8*E*). Both R878 and D886 are in the C-terminal 1/3 and are not directly involved in the assembly of this trimer (Fig. 8*E*). Thus, both R878Δ and D886G mutations most likely do not affect the formation of the coiled-coil trimer of PC2. We have previously found that even deleting the entire C-terminal region of this coiled-coil domain has no such effect (16). Consistently, we did not see channel function change after introducing these two mutations in PC2_F604P (Fig. 8*B*). It is worth noticing that D866G was previously proposed to cause an mRNA splicing variant with frameshift (33). It could explain the possible cause of ADPKD with this mutation. At the same time, the R878Δ mutation will likely interfere with the coiled-coil interaction between PC1 and PC2, which is discussed in the Discussion below. No structural information is available for R798, T931, and S949. How T931M reduces the channel current of PC2_F604P is presently unknown.

## Discussion

Understanding how pathogenic mutations affect the PC2 channel activity helps us gain insight into the structure and function of PC2 and how malfunction of this channel plays its role in ADPKD. However, due to the lack of knowledge on PC2 channel activation, it has been challenging to carry out the related study. The F604P GOF mutation found in our previous work leads to constitutive activation of PC2 and allows us to record reliable and robust current from this ion channel (10). With the PC2_F604P mutant channel, we were able to systematically test the effects of 31 single point mutations, including 26 clinically pathogenic mutations, on PC2 channel activity. Since F604P mutation-caused conformational changes in PC2, especially that in the channel pore region and the gate, is similar to what was found in the gating process of other TRP channels, we believe the structure of PC2_F604P mimics wildtype PC2 in a natural open status. Thus, the results we obtained in this study will help us gain insight into how these pathogenic mutations impact WT PC2 channel activity. At the same time, a drawback of using a GOF mutant is that its activation has bypassed the channel gating process. Thus, if a mutation affects gating, we will not be able to detect this effect. However, due to the challenge of recording wildtype PC2 channel activity, the GOF mutant channel is still the best choice we have for the current study.

Our results show that these pathogenic mutations cause various effects on the channel activity of PC2_F604P. The TOP domain is a hotspot of known pathogenic point mutations of PC2. The majority of these mutations, 17 out of 26, occur in the TOP domain (Fig. 1). Within these 17 mutants, 12 of them abolish or significantly reduce the channel activity of P2C_F604P without affecting the plasma membrane trafficking of the channel in oocytes (Figure 2, Figure 3, Figure 4, Figure 5). We predicted that most of these mutations will lead to significant local conformational changes in TOP structure by disrupting key interactions within or between TOP domains. These results demonstrate the essential role of the TOP domain in PC2 channel function. Our results also show that all seven pathogenic point mutations in the transmembrane domains, S4-S5 linker, and the pore region abolish channel function (Figs. 6 and 7). These results are consistent with the assumption that the transmembrane domains, especially S3-S5 and the S4-S5 linker, are critical for channel structure and gating in TRP channels and voltage-gated ion channels (32, 47), while the pore region is essential for ion conductance. Most C-terminal tail mutations we have tested in this study, including two clinical pathogenic mutations R878Δ and D886G, led to relatively mild effects, if any, on PC2_F604P channel activity. How these mutations, as well as the TOP domain mutations that do not affect channel activity, lead to ADPKD cannot be explained by our current results. Their possible pathogenic mechanisms may include (1) affecting the gating of the wildtype PC2 channel; (2) interfering with the trafficking of PC2 *in vivo*, including that to primary cilia; (3) leading to abnormal alternative splicing of PKD2 RNA as shown in previous studies (33, 48, 49); and (4) leading to dysfunction of the heteromeric PC1/PC2 channel, which was not tested in this study. It is also possible that one mutation causes a consequence that is a combination of different effects.

The effects of some of these PC2 pathogenic mutations on PC2 function have been tested in previous studies. Among these mutations, D511V in S3 has been widely reported to abolish the PC2 channel function (10, 11, 34, 36, 39, 45, 50, 51), which is consistent with our finding here (Fig. 6). Previously, Vien *et al*. tested the effects of a group of pathogenic mutations in the TOP domain (R322W, R322Q, R325P, R325Q, and C331S) on PC2 channel activity in primary cilia (31). They found that all these five tested mutations did not affect the ciliary trafficking of PC2. However, they all abolished PC2 current when tested within a voltage range from −100 to +100 mV, due to the large positive shift of the voltage threshold in the mutants (31). In our tests, we also found C331S almost completely abolished PC2_F604P current, and R322Q significantly reduced the current (Figs. 2 and 3). However, R322W, R325P, and R325Q mutants had similar channel activity as PC2_F604P (Figs. 2 and 3). The results suggest a possibility that although these mutations interfere with the voltage dependence of the channel activity, it is not detectable anymore once the channel is opened by the GOF mutation F604P. It is worth noticing though that the voltage-dependent gating of the PC2 channel was not seen when it was expressed in either *Xenopus* oocyte or HEK 293T cells (10, 16, 25); thus, this property may be related to the microenvironment in cilia. A previous study reported that the PC2-W414G mutant did not go to primary cilia when expressed in LLC-PK1 cells but gave rise to similar channel activity as the WT channel in a single-channel recording using bilayers made from ER-enriched vesicles (52). Here, we found W414G abolishes PC2_F604P current (Fig. 4). This inconsistency may be caused by different methods and systems used in the recording and needs to be further investigated. The missense mutation c.1320G > T (R440S) in the TOP domain was previously found to interfere with normal RNA splicing of PC2 and lead to more production of a variant that is missing exon 6 (48). Our result here shows that besides the effect on splicing, R440S also causes a reduction of PC2 ion channel function (Fig. 3).

How these PC2 pathogenic mutations affect the function of the PC1/PC2 heteromeric channel is worth further investigation. Since the TOP domain is directly involved in the assembly of the PC1/PC2 complex, we assume that the mutations that happen at the interface of PC2 TOP domains, such as the mutations in finger 1, will potentially also change the assembly and function of the heteromeric channel. This may also be true for mutations located in the pore region. We have discussed above that R878Δ and D886G do not affect the coiled-coil trimer formation of the C-terminal tail of PC2 since they are not in the tightly bundled portion of this trimer. However, both our experimental and computational modeling results suggested that the C-terminal loose structure of this coiled-coil trimer forms the major interface for the association of the PC1 coiled-coil domain (Fig. 8*F*) (15, 16). In our model, the PC1/PC2 coiled-coil domain complex shows a di-trimer configuration, with an upstream dimer formed by all three PC2 helices and a downstream trimer formed by one PC1 helix and two PC2 helices (Fig. 8*F*) (15). The K876/R877/R878/E879 charge cluster increases the flexibility of the C-terminal portion of the PC2 helix and facilitates the formation of the trimer with PC1. Also, R877 and R878 directly interact with E4239 and D4234, respectively, and are essential for the assembly of this complex (Fig. 8*F*) (15). Double mutation R877G/R878G almost completely abolished the binding between PC1 and PC2 coiled-coil domains (15). The deletion mutation R878Δ will not only disrupt the interaction at this site but also induce a rotation of the downstream region of the PC2 coiled-coil helix and disturb the downstream hydrophobic interaction that was shown to be critical for the assembly of the PC1/PC2 coiled-coil complex in our model (15). Together, these findings help us gain insight into a hypothesis that some PC2 C-terminal pathogenic mutations cause ADPKD likely through disrupting the assembly of PC1/PC2 complex, instead of PC2 homotetramer.

## Experimental procedures

### cDNA constructs and cloning

Human TRPP2 cDNAs (National Center for Biotechnology Information accession no. U50928) were cloned into a modified pGEMHE vector for *in vitro* transcription. Mutations were generated by PCR, and all constructs were confirmed by sequencing.

### Electrophysiology

RNAs were synthesized *in vitro* and injected into *Xenopus laevis* oocytes (30 ng RNA/oocyte). After injection, oocytes were incubated at 18 °C for 2 to 3 days, and the TEVC method was used to record whole-cell currents. All recordings have been repeated with at least three batches of oocytes, and most of them have been completed additional times. Unless otherwise indicated, oocytes were recorded at room temperature in a bath solution containing 100 mM NaCl, 2 mM CaCl_2_, and 2 mM HEPES, pH 7.5.

A standard TEVC protocol includes holding oocytes at 0 mV and measuring the current-voltage (I-V) relationships by applying 80 ms voltage steps from −80 to +60 mV in 10 mV increments.

### Oocyte lysate preparation

The protein expression of all mutations tested in this study has been confirmed by Western blot. All Western blots were repeated at least two times. To prepare oocyte lysate samples, oocytes were collected after TEVC recording and washed twice with cold OR2 solution (82.4 mM NaCl, 2.5 mM KCl, 1 mM MgCl_2_, 10 mM HEPES, pH 7.6). They were then incubated in a lysis solution containing 1x PBS, 1 mM EDTA, 10% glycerol, 1% n-Dodecyl- β-D-maltoside, and 1/50 (v/v) Protease Inhibitor Cocktail (Sigma Aldrich). Ten microliter of lysis solution was used per oocyte. Oocytes were homogenized by passing through a 25-G needle 10 times, and lysates were incubated by rotating at 4 °C for 1 h. After centrifuging for 30 min at 10,000*g*, supernatants were collected.

### Oocyte surface protein purification with biotinylation

Proteins expressed on *Xenopus* oocyte plasma membrane were detected at least twice with the Pierce Cell Surface Biotinylation and Isolation kit following a modified protocol described previously (53). Briefly, 2 to 3 days after RNA injection, oocytes (40 per group) were collected and washed twice with cold OR2 solution (82.4 mM NaCl, 2.5 mM KCl, 1 mM MgCl_2_, 10 mM HEPES, pH 7.6). Oocytes were then incubated with 0.4 mg/ml sulfo-NHS-SS-biotin in ice-cold OR2 at 4 °C for 30 min. The reaction was then quenched, and oocytes were washed, following the manufacturer’s protocol. Oocytes were lysed, and supernatants were collected as described above. After centrifuging for 30 min at 10,000*g*, supernatants were collected. Lysates were mixed with NeutrAvidin Agarose at 4 °C overnight. After beads were washed, proteins were eluted with 50 mM DTT at 37 °C for 30 min. Eluted samples were analyzed by SDS–PAGE and Western blot.

### SDS-PAGE and Western blot

Oocyte lysate and surface biotinylation samples were run on 4 to 12% Bolt Bis-Tris Plus gels (Thermo Fisher Scientific) and transferred to polyvinylidene fluoride membrane. Mouse monoclonal anti-PC2 antibody (YCE2, Santa Cruz Biotechnology), mouse monoclonal anti-β-actin antibody (GenScript), and IRDye 680RD goat-anti-mouse IgG secondary antibody (Li-COR) were used. Blot signals were visualized with LI-COR Odyssey.

### Structural graphics

The structural graphics of TRPP2 shown in all figures were prepared with the software PyMOL (The PyMOL Molecular Graphics System).

### Statistical analysis

Electrophysiology data were analyzed with Excel or GraphPad Prism 8, and statistical significance was determined by an unpaired, two-tailed Student’s *t* test. The currents of F604P and the other new mutants were normalized to the mean of F604P currents recorded from the same batch of oocytes, and the results of each individual mutant was compared to that of F604P in *t* test. *t* test results of *p*< 0.05 were considered statistically significant (differences *p*< 0.05 denoted by ∗*p*< 0.01 denoted by ∗∗, and *p*< 0.001 denoted by ∗∗∗). Results are presented as mean ± SD. Raw data used in bar graphs and *t*-tests are included in Supporting information. One recorded current from W280R and another one from T448K were removed during data analysis since they are unreasonably much larger than all other currents from the same mutant, possibly due to membrane leakage.

### Animal use

Frogs care and experimental protocols were conducted upon approval of the Institutional Animal Care and Use Committee (IACUC) at St John’s University.

## Data availability

All data are contained within the manuscript.

## Supporting information

This article contains supporting information.

## Conflict of interest

The authors declare that they have no conflicts of interest with the contents of this article.
